# Gastrointestinal Beriberi Presenting With Chronic Diarrhea: A Case Report

**DOI:** 10.7759/cureus.99095

**Published:** 2025-12-13

**Authors:** Ryoma Ouchida, Masataka Kudo

**Affiliations:** 1 Department of General Internal Medicine, Iizuka Hospital, Iizuka, JPN

**Keywords:** beriberi, malnutrition, persistent diarrhea, post-gastrectomy, thiamine or vitamin b1 deficiency, weight loss

## Abstract

Gastrointestinal beriberi is an underrecognized yet important manifestation of thiamine deficiency that may occur even in individuals without a history of chronic alcohol use. It often presents with nonspecific upper gastrointestinal symptoms, leading to diagnostic delay. The case of a woman in her 60s with a history of distal gastrectomy who developed persistent anorexia and intractable diarrhea over four months, resulting in multiple hospitalizations, is presented. Despite comprehensive evaluations, including blood tests, CT scans, stool analyses, endoscopy, and biopsies, no definitive cause was identified. Furthermore, she was not taking any medications that might have contributed to the diarrhea. She had no recent alcohol misuse, neurological deficits, or lactic acidosis, which are classical features typically associated with thiamine deficiency. Given her surgical history and ongoing symptoms, thiamine deficiency was suspected. A serum thiamine level measured on hospital day one was low at 14 ng/ml (reference range 26-58), which was consistent with thiamine deficiency. Oral thiamine led to the rapid resolution of symptoms within one week, and we therefore diagnosed thiamine deficiency. This case highlights gastrointestinal beriberi as an early, isolated manifestation. Diarrhea was the predominant symptom, a less-reported feature. Early recognition and treatment may prevent serious complications such as Wernicke’s encephalopathy, even without classic risk factors.

## Introduction

Thiamine deficiency remains a significant public health concern in developing countries, particularly in low-income regions of Asia and Africa [1]. Although less common in industrialized nations, it still occurs, most frequently among individuals with chronic alcohol use. However, non-alcoholic individuals are also at risk, especially those with malignancies or a history of gastrointestinal surgery [2].

Clinically, thiamine deficiency is classified into dry beriberi, characterized by peripheral neuropathy and Wernicke’s encephalopathy, and wet beriberi, which presents as high-output heart failure. In contrast to these neurologic and cardiovascular forms, gastrointestinal beriberi is defined as thiamine deficiency that primarily affects the digestive system, presenting with nonspecific upper gastrointestinal symptoms such as abdominal pain, nausea, vomiting, and anorexia. These symptoms may be refractory to standard therapies and improve dramatically following thiamine administration. It has been proposed as a prodromal phase of Wernicke’s encephalopathy [3], highlighting the importance of early recognition and treatment. However, in the absence of neurological signs or lactic acidosis, thiamine deficiency may be overlooked when gastrointestinal symptoms appear in isolation.

Although diarrhea has occasionally been reported in association with thiamine deficiency [4], detailed case reports remain scarce, and diarrhea is rarely reported as the predominant clinical manifestation. To date, our comprehensive literature search has identified only seven published case reports of gastrointestinal beriberi and a single prospective observational study of thiamine-responsive upper gastrointestinal upset (“gastric beriberi”); in these reports, patients have mainly presented with acute gastrointestinal symptoms such as nausea, vomiting, and abdominal pain, and chronic diarrhea has not been highlighted as a predominant or persistent manifestation [3,5-9]. As a result, clinicians may not immediately consider thiamine deficiency in patients presenting with diarrhea, which can lead to diagnostic challenges and delays in initiating appropriate treatment. We report a case of gastrointestinal beriberi presenting predominantly with chronic diarrhea. This case highlights a significant diagnostic pitfall and emphasizes the importance of considering thiamine deficiency, even when gastrointestinal symptoms occur in isolation.

## Case presentation

**Figure 1:**
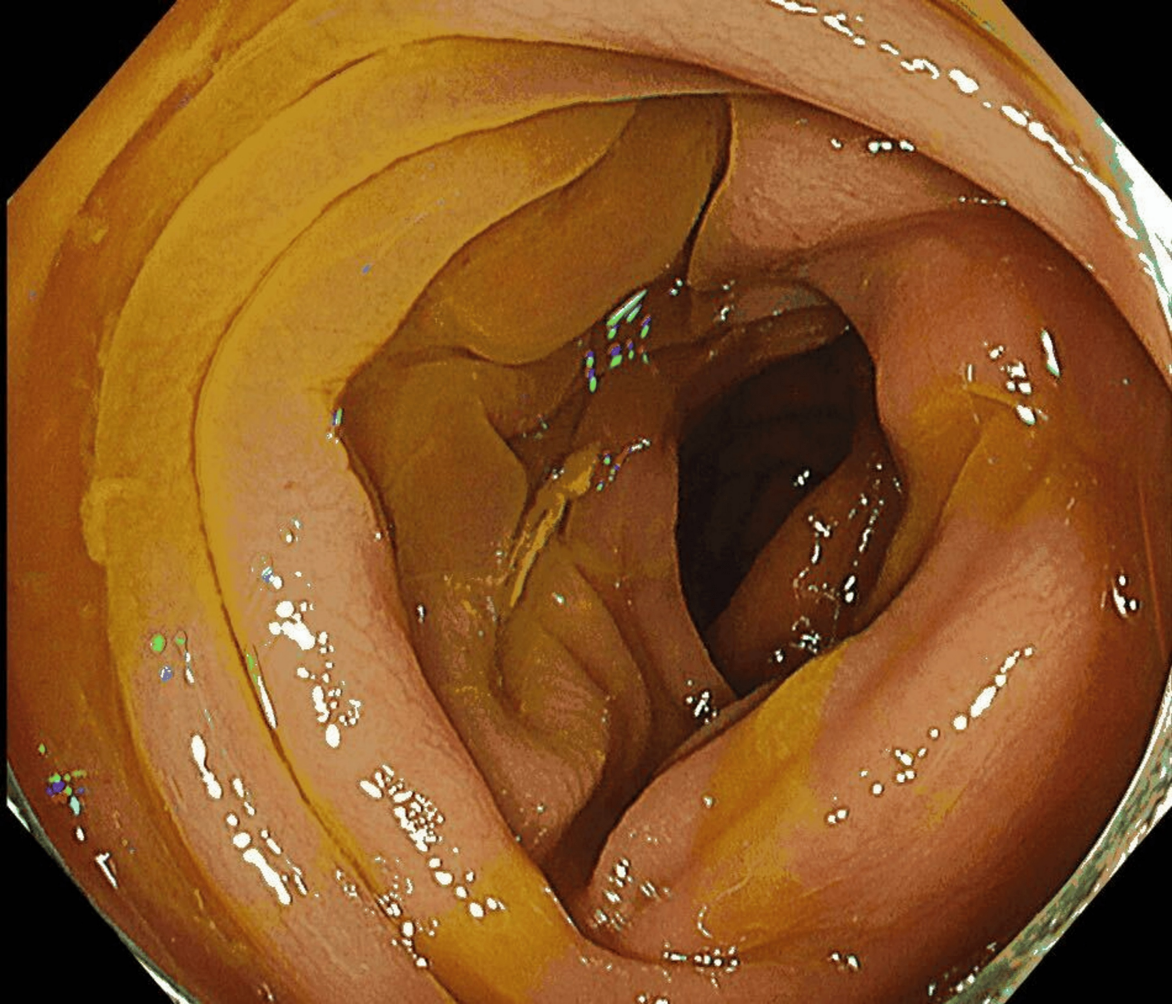
Colonoscopy findings Colonoscopy revealed diffuse mucosal edema throughout the entire colon.

A woman in her 60s, with a history of distal gastrectomy and Billroth I reconstruction for gastric cancer 18 years earlier and primary biliary cholangitis, presented with nausea, significantly reduced oral intake, and a four-month history of anorexia. Although she did not have any particular food preferences or selective eating patterns, during this period, she was able to consume only approximately 0-20% of her usual meals. The quality of her diet was similar to her pre-illness intake, but the overall caloric intake was markedly reduced. She had an uneventful postoperative course, and long-term surgical follow-up for postoperative complications and nutritional monitoring had already been completed. She had a history of heavy alcohol use until five years earlier and had not consumed alcohol since that time. During this period, she experienced a 10-kg weight loss, from a baseline of 87 kg to 77 kg, and was hospitalized two months before her current presentation. At that time, blood tests and contrast-enhanced computed tomography showed no organic pathology. Potential causes of marked weight loss-including malignant, endocrine, neuromuscular, inflammatory, and chronic organ failure conditions such as heart failure and chronic obstructive pulmonary disease-were considered and ruled out. The weight loss was attributed to psychosocial stress within the family. Following the resolution of these family conflicts, she was able to resume minimal oral intake and was subsequently discharged. Five days after discharge, she developed recurrent episodes of diarrhea accompanied by abdominal pain, which again made oral intake difficult, leading to a second hospitalization. Colonoscopy showed diffuse colonic edema (Figure 1), and nonspecific colitis was diagnosed. The biopsy revealed only mild infiltration of inflammatory cells, including a small number of neutrophils, with prominent interstitial edema. The diarrhea followed a relapsing-remitting pattern. Although symptoms improved intermittently, the condition did not resolve completely, and she was discharged once again. However, her diarrhea persisted and worsened over the following weeks, resulting in a third hospitalization. During the following two months, her weight decreased by an additional 13 kg despite supportive measures, and treatment for suspected post-infectious irritable bowel syndrome (PI-IBS) with ramosetron was started.

On admission, her Glasgow Coma Scale (GCS) score was 15 (E4V5M6), indicating that she opened her eyes spontaneously, was fully oriented and communicative, and responded appropriately to commands. Vital signs were stable, with a blood pressure of 110/67 mmHg, heart rate of 78 bpm, temperature of 36.5 °C, and oxygen saturation of 99%. Physical examination showed lower abdominal tenderness; the rest of the findings were unremarkable. Neurologically, there were no abnormalities in ocular movement, coordination, or sensorimotor function. Laboratory tests revealed mild hypokalemia and hypoalbuminemia; however, bilirubin and GGT levels remained largely unchanged compared to previous measurements (Table 1). Serum lactate was within normal limits on admission (1.72 mmol/L) and remained normal on the following day (1.93 mmol/L); no further measurements were obtained thereafter.

**Table 1:** Laboratory findings

Parameters	Parameters	Reference range
White blood cell count (/uL)	6,750	4000-11,000
Hemoglobin (g/dL)	14.3	13-18
Platelet ( x 10^3 /uL)	213	158-348
Alanine aminotransferase (IU/L)	21	<65
Aspartate aminotransferase (IU/L)	52	<50
Alkaline phosphatase (IU/L)	67	40-129
Gamma-glutamyl transferase (IU/L)	81	8-61
Total bilirubine (mg/dL)	2.4	0.4-1.5
Sodium (mmol/L)	141	136-145
Potassium (mmol/L)	2.6	3.5-4.6
Chloride (mmol/L)	95	101-108
Blood urea nitrogen (mg/dL)	5	8-20
Creatinine (mg/dL)	0.56	0.65-1.07
C-reactive protein (mg/L)	0.8	<5
Albumin (g/L)	26	35-50
Thyroid stimulating hormone (μIU/mL)	1.72	0.61-4.23
HbA1c (%)	5.8	4.9-6.0
Antinuclear antibody	negative	negative

**Figure 2:**
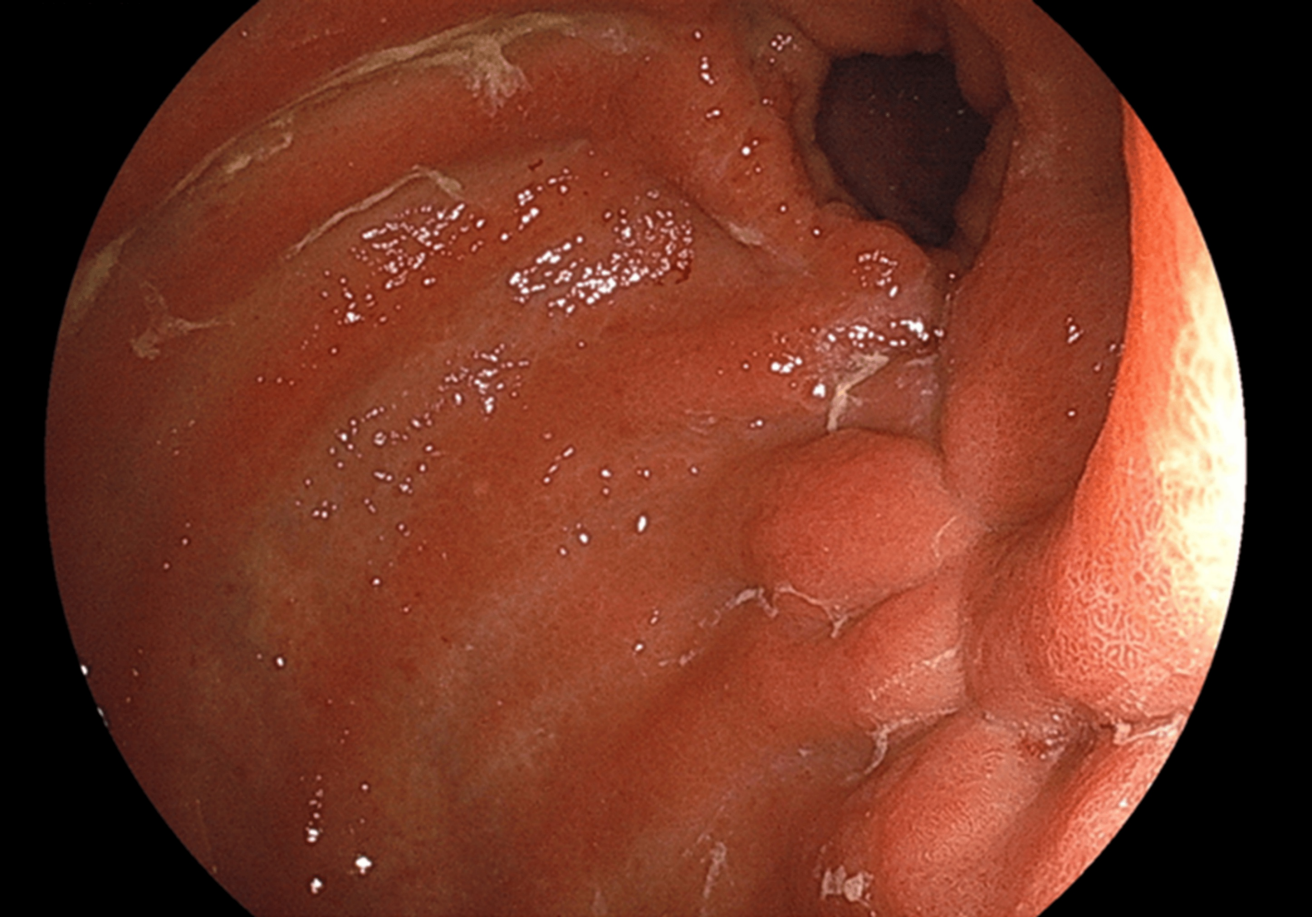
Upper gastrointestinal endoscopy findings Upper gastrointestinal endoscopy revealed a post-Billroth I reconstruction state. The anastomosis site and other regions showed no signs of masses or mucosal abnormalities, indicating a normal post-surgical condition.

**Figure 3:**
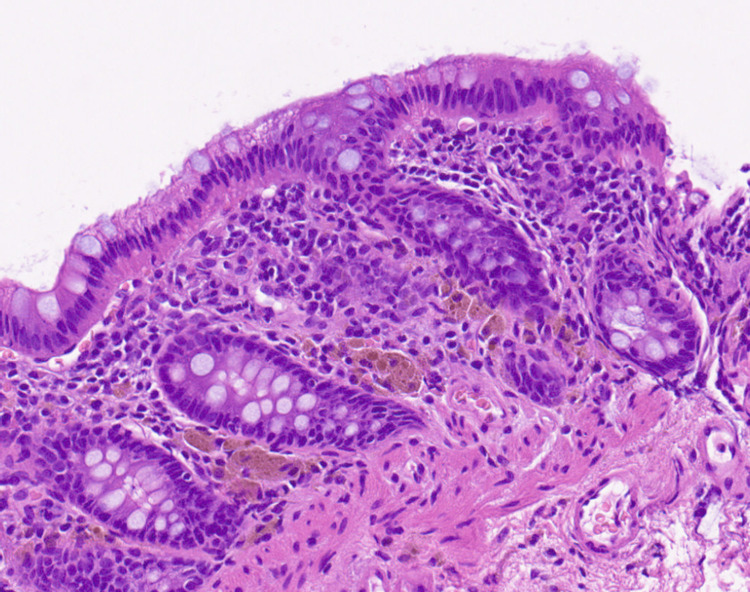
Pathological findings from random colonic biopsies. H&E; magnification, ×20 The biopsy showed no evidence of specific inflammation or malignancy. No villous atrophy or other subtle mucosal changes were observed.

Given the patient’s chronic diarrhea and weight loss, an evaluation for organic disease was prioritized. Infectious causes were excluded by negative stool cultures and absence of *Clostridioides difficile* antigen. Upper gastrointestinal endoscopy showed no abnormalities (Figure 2), and colonoscopy with random biopsies showed diffuse edema without histological evidence of inflammatory bowel disease, amyloidosis, or eosinophilic infiltration (Figure 3). Despite these evaluations, no definitive organic etiology was identified. During her inpatient supportive care, the patient’s diarrhea showed slight improvement without fully resolving, and she was subsequently discharged. In light of the persistent symptoms after discharge, alternative underlying causes were considered.

Although diarrhea is an uncommon manifestation, her marked weight loss, poor oral intake, and history of distal gastrectomy with Billroth I reconstruction, and low albumin level, indicative of chronic malnutrition, led us to consider thiamine deficiency as a likely contributing factor. Serum thiamine levels were markedly decreased to 14 ng/mL. Oral thiamine supplementation reduced the frequency of diarrhea by about 50% within one week and led to an almost complete resolution of symptoms by four weeks.

## Discussion

This case illustrates a presentation of gastrointestinal beriberi in a non-alcoholic patient, characterized by anorexia and persistent diarrhea in the absence of neurological or metabolic signs typically associated with thiamine deficiency. Although the patient had a clinical history suggestive of malnutrition, the diagnosis of thiamine deficiency was delayed. Several factors contributed to this diagnostic challenge: chronic diarrhea was the predominant symptom; few additional findings indicated thiamine deficiency; and Billroth I reconstruction is a surgical procedure generally considered to carry a relatively lower risk of thiamine deficiency than other types of gastric surgery. Table 2 summarizes previously reported cases of gastrointestinal beriberi, including both alcoholic and non-alcoholic patients. As shown, almost all reported cases presented with upper gastrointestinal symptoms such as nausea, vomiting, or anorexia, and were accompanied by either lactic acidosis or neurological findings suggestive of Wernicke’s encephalopathy. Although there has been a report of a thiamine-deficient patient presenting with upper gastrointestinal symptoms, diarrhea, and lactic acidosis [10], that case was not recognized as gastrointestinal beriberi, and diarrhea was not described as a thiamine-deficiency manifestation nor reported to improve with thiamine therapy; therefore, it was not included in Table 2. The present case also had upper gastrointestinal symptoms that improved after thiamine supplementation. However, it was distinguished by persistent diarrhea in the absence of metabolic or neurological abnormalities, representing an earlier and atypical presentation of gastrointestinal beriberi that has rarely been described in the literature.

**Table 2:** Reported cases of gastrointestinal beriberi including the present case

Case	Age (years)	Sex	Background disease/condition	Gastrointestinal symptoms	Neurological symptoms	Lactate level (mmol/L, normal range 0.5–2.0)
Present case	60	F	Non-alcoholic; post-gastrectomy	Nausea, vomiting, abdominal pain, and diarrhea	No symptoms	1.72
Prakash, 2018 [3]	38	M	Non-alcoholic	Abdominal fullness, indigestion, abdominal pain, nausea, and decreased appetite	Headache, vertigo, gaze-evoked nystagmus, impaired finger-nose testing, and impaired tandem walk	Not reported
	21	F	Non-alcoholic; pulmonary tuberculosis	Anorexia, epigastric discomfort, and nausea	Slurring of speech, vertigo, gaze-evoked nystagmus, abnormal finger-nose testing, and gait ataxia	Not reported
Donnino, 2004 [5]	57	M	Alcohol use	Nausea, vomiting, and abdominal pain	Not reported	27.0
	55	F	Alcohol use	Abdominal pain	Not reported	19.9
Vu, 2019 [6]	81	M	Non-alcoholic; rheumatoid arthritis	Abdominal pain, nausea, and weakness	Not reported	14.4
Hayashi, 2024 [7]	82	F	Non-alcoholic; uterine cancer (stage IIIB)	Nausea and complained of decreased dietary intake	Myoclonus on the left side, difficulty speaking, slurred speech, and drooping of the left corner of the mouth, with no consciousness disturbance	Not reported
Duca, 2016 [8]	30	M	Non-alcoholic	Intermittent abdominal pain, nausea, and non-bloody vomiting	Not reported	2.6–8.0

First, the predominance of chronic diarrhea in this case is considered to have compounded the diagnostic challenge. Whereas previous reports have emphasized upper gastrointestinal symptoms such as nausea, vomiting, or anorexia as the main features [11], these symptoms were also present in the current case; however, the concurrent presence of diarrhea distinguishes this presentation from earlier cases [3,5-8,11]. Although not explicitly diagnosed as gastrointestinal beriberi, there are case reports of thiamine deficiency presenting with diarrhea and lactic acidosis [10], as well as technical reports noting that diarrhea may occur in gastrointestinal beriberi [4]. Physiologically, thiamine deficiency is thought to cause autonomic dysfunction [12] and alterations in the gut microbiota, both of which may contribute to the development of diarrhea [13]. Most previously reported cases have focused on upper gastrointestinal symptoms; however, clinicians should be aware that gastrointestinal beriberi can present with persistent diarrhea as the predominant symptom.

Second, since the patient did not currently consume alcohol and exhibited neither neurological findings consistent with Wernicke’s encephalopathy nor lactic acidosis, thiamine deficiency was not initially suspected. A stepwise evaluation ruled out infectious etiologies, as well as inflammatory, infiltrative, and eosinophilic colonic disorders, yet her gastrointestinal symptoms persisted, prompting consideration of malabsorption and micronutrient deficiencies. Gastrointestinal beriberi is most often observed in patients with alcohol use disorder presenting with upper gastrointestinal symptoms [5], whereas in non-alcoholic patients, thiamine deficiency was most commonly diagnosed retrospectively, after the development of elevated lactate levels or neurological manifestations consistent with Wernicke’s encephalopathy [3,6-8,11]. The mechanisms underlying the differing patterns of symptom manifestation in thiamine deficiency are not fully understood [4], and it is well established that the neurological features of Wernicke’s encephalopathy can vary widely [14]. Consequently, it remains unclear to what degree of thiamine depletion neurological abnormalities become clinically apparent. In the case series reported by Prakash et al., some patients who eventually developed Wernicke’s encephalopathy had already exhibited gastrointestinal symptoms before the onset of neurological manifestations, suggesting that gastrointestinal beriberi may represent an early stage of thiamine deficiency. These observations suggest that, when gastrointestinal symptoms occur in isolation, diagnosis is frequently delayed, and such delays may contribute to the development of Wernicke’s encephalopathy [3]. In the present case, however, thiamine deficiency was suspected and treatment was initiated before the onset of elevated lactate levels or neurological manifestations, which may have averted progression to severe complications.

Third, thiamine deficiency is most commonly observed after procedures that bypass the duodenum (e.g., total gastrectomy, Billroth II, Roux-en-Y), as thiamine is primarily absorbed in the duodenum and proximal jejunum. Consequently, bypassing these segments significantly impairs absorption and increases the risk of deficiency. However, subclinical deficiency has been documented in up to 30% of gastrectomy patients overall [15]. In addition to the bypassing of primary absorption sites, several other mechanisms have been proposed to contribute to thiamine deficiency after gastrectomy, including reduced oral intake, malabsorption, and small intestinal bacterial overgrowth [16]. Collectively, these factors suggest that patients with Billroth I reconstruction also remain at risk, particularly in the presence of malnutrition or persistent gastrointestinal symptoms. Moreover, delayed presentations occurring years after gastrectomy, following various types of reconstructive surgery [15,17], have been reported, highlighting the importance of long-term monitoring in post-gastrectomy patients.

This case offers valuable insight into the risk of delayed diagnosis in such presentations, and we recommend that gastrointestinal beriberi be considered in patients with unexplained gastrointestinal symptoms, even in the absence of typical risk factors. However, serum thiamine assays are not always readily available and, even when they can be performed, the measured levels may show considerable variability and can be affected by sampling and handling conditions, limiting their reliability as a diagnostic marker [18]. Therefore, when clinical suspicion is high, a therapeutic trial of thiamine should be initiated even if serum thiamine levels are within the reference range or cannot be measured.

## Conclusions

Thiamine deficiency remains an underrecognized condition that is nevertheless clinically significant, particularly in patients with a history of gastrointestinal surgery or malnutrition. Gastrointestinal beriberi often presents with nonspecific abdominal symptoms and may be overlooked in the absence of neurological signs. Importantly, neurological manifestations of thiamine deficiency often appear later in the disease course. As a result, early presentations with isolated gastrointestinal symptoms are particularly prone to being missed. In patients with unexplained gastrointestinal complaints, particularly diarrhea, without definitive imaging or endoscopic findings, clinicians should maintain a high index of suspicion for thiamine deficiency. Empirical thiamine supplementation may lead to rapid symptom improvement, as observed in the present case, in which gastrointestinal symptoms improved within one week of treatment, and may prevent progression to neurological complications such as Wernicke’s encephalopathy. Because thiamine supplementation is generally low risk, it can be used empirically in uncertain clinical scenarios, serving both diagnostic and therapeutic purposes.
